# Supplementary material for: Calibrating a Comprehensive Immune Age Metric to Analyze the Cross Sectional Age-Related Decline in Cardiorespiratory Fitness
Source: Biology (Basel). 2022 Oct 27;11(11):1576. doi: 10.3390/biology11111576 (PMC9687950; doi:10.3390/biology11111576)
Supplement: Supplementary file 1 [file biology-11-01576-s001.zip › biology-1958052-supplementary.pdf]

Supplemental Material

to

Calibrating a Comprehensive Immune Age Metric to Analyze the Cross Sectional Age-Related Decline in Cardiorespiratory Fitness

Supplemental Material 1

1 Supplemental Tables 2

Table S1: Panel description and material for PBMC staining. 2

Table S2: List of 65 variables with cellular frequencies. 3

Table S3: Fitted principal component regression model predicting the IMM-AGE metric. 4

Table S4: Comparison of in multiple logistic regression models including both chronological age and immune age metrics as predictors. 5

Table S5: Comparison of different age metrics (chronological age and immunosenescence biomarkers) predicting low CRF in separate models. 6

2 Supplemental Figures 7

Figure S1: Gating strategy with general overview of lymphocyte and monocyte subpopulations 7

Figure S2: Gating strategy for T cell subpopulations. 8

Figure S3: Correlation between IMM-AGE and 65 relative cellular frequencies 9

Figure S4: Comparison of 16 cellular frequencies between the DVS and IMM-AGE study 10

Figure S5: Comparison of linear regression lines for twelve candidate predictors related to age between the DVS and IMM-AGE study 11

3 Supplemental Code 12

Code S1: R script accessing IMM-AGE data and calculating IMMAX. 12

4 Abbreviations 18

5 References 19

1 Supplemental Tables

Table S1: Panel description and material for PBMC staining.

Panel description, antigens, antibody clones and coupled fluorochromes, distributors and antibody dilution used to stain 0.2 × 10^6 PBMC are listed

panel	*antigen*	*clone*	*fluorochrome*	*company*	*dilution 1/x*
lymphocytes / monocytes	CD19	HIB19	BV421	BD Horizon™	200
	CD3	UCHT1	BV510	BD Horizon™	400
	live / dead		zombie Yellow	Biolegend	1000
	CD16	3G8	FITC	BD Pharmingen™	200
	CD14	MϕP9	PE	BD Pharmingen™	500
	CD64	10.1	PE-Cy™7	BD Pharmingen™	200
	CD56	B159	APC	BD Pharmingen™	50
	CD45	HI30	Alexa Fluor® 700	BD Pharmingen™	500
NK + T cells	CD3	UCHT1	BV510	BD Horizon™	400
	live / dead		zombie Yellow	Biolegend	1000
	CD8	RPA-T8	FITC	BD Pharmingen™	200
	CD28	CD28.2	PerCP-Cy™5.5	BD Pharmingen™	100
	CD57	NK-1	PE	BD Pharmingen™	800
	CD56	B159	PE-CF594	BD Pharmingen™	100
	CD197 (CCR7)	150503	Alexa Fluor® 647	BD Pharmingen™	50
	CD4	RPA-T4	APC-H7	BD Pharmingen™	100
	CD45RA	HI100	Alexa Fluor® 700	BD Pharmingen™	400

Table S2: List of 65 variables with cellular frequencies.

List of 65 variables with cellular frequencies reported in the supplementary Table sTable5 (https://static-content.springer.com/esm/art%3A10.1038%2Fs41591-019-0381-y/MediaObjects/41591_2019_381_MOESM3_ESM.xlsx) of the original IMM-AGE study (Alpert et al. 2019) with grey shaded entries indicating candidate items potentially shared with the DVS data. Bold codes indicate the abbreviations used in figures and tables.

basophils	HLADRnegCD38posCD4pos.T.cells
B.cells (***B***)	HLADRposCD38posCD4pos.T.cells
NK.cells (***NK***)	HLADRnegCD38posCD8pos.T.cells
T.cells (***T***)	HLADRposCD38posCD8pos.T.cells
CD161negCD45RApos.Tregs	HLADRpos.NK.cells
CD161posCD45RApos.Tregs	ICOSposCD4pos.T.cell
CD161posCD4pos.T.cells	ICOSposCD8pos.T.cell
CD161posCD8pos.T.cells	IgDposCD27neg.B.cells
CD161pos.NK.cells	IgDposCD27pos.B.cells
CD16negCD56bright.NK.cells	lymphocytes
CD16pos.monocytes (***CD16 mono***)	mDCs
CD27posCD8pos.T.cells	memory.B.cells
CD28negCD8pos.T.cells (***CD8 CD28-***)	monocytes
CD4posCD27pos.T.cells	naive.B.cells
CD4posCD28neg.T.cells (***CD4 CD28-***)	NKT.cells
CD4pos.T.cells (***CD4***)	PD1posCD4pos.T.cells
CD57posCD4pos.T.cells	PD1posCD8pos.T.cells
CD57posCD8pos.T.cells	pDCs
CD57pos.NK.cells	plasmablasts
CD85jposCD4pos.T.cells	CXCR5+ CD4+T
CD85jposCD8pos.T.cells	CXCR5+ CD8pos.T.cells
CD8pos.T.cells (***CD8***)	Th17 CXCR5-CD4pos.T.cells
CD94posCD4pos.T.cells	Th17 CXCR5+ CD4pos.T.cells
CD94posCD8pos.T.cells	CXCR3negCCR6pos CXCR5+CD8pos.T.cells
CD94pos.NK.cells	Th1.non.TFH.CD4pos.T.cells
central.memory.CD4pos.T.cells (***CD4 central mem***)[a]	Th1.TFH.CD4pos.T.cells
effector.memory.CD4pos.T.cells (***CD4 effector mem***)[a]	CXCR3posCCR6neg CXCR5+CD8pos.T.cells
naive.CD4pos.T.cells (***CD4 naive***)	Th2.non.TFH.CD4pos.T.cells
central.memory.CD8pos.T.cells (***CD8 central mem***)[a]	Th2.TFH.CD4pos.T.cells
effector.memory.CD8pos.T.cells (***CD8 effector mem***)[a]	CXCR3negCCR6neg CXCR5+ CD8pos.T.cells
naive.CD8pos.T.cells (***CD8 naive***)	transitional.B.cells
effector.CD4pos.T.cells	Tregs
effector.CD8pos.T.cells	

[a] Note that CD4 and CD8 memory cell frequencies (***CD4 mem***, ***CD8 mem***) were computed as added effector and central memory frequencies from the corresponding subsets

Table S3: Fitted principal component regression model predicting the IMM-AGE metric.

Sample standard deviations (*SD$_i$*) of predictors (*x$_i$*) from the IMM-AGE study (Alpert et al. 2019) and coefficients (*ß$_i$*) of the principal component regression model predicting the logit-transformed IMM-AGE metric $logit.IMM.AGE = \beta_0 + \sum_{i=1}^{5} \beta_i \times x_i / SD_i$, which is then used to calculate the approximation as $IMMAX = \exp(logit.IMM.AGE)/(1 + \exp(logit.IMM.AGE))$.

Index i	***Predictor x_i***	***SD_i***	***β_i***
0	(Intercept)	–	0.0248
1	log CD8 mem/naive	1.6800	0.3962
2	logit CD8 CD28-	1.4338	0.3267
3	log CD4 mem/naive	1.0774	0.2549
4	log NK/T	1.0540	0.1623
5	log CD4/CD8	0.6836	0.0437

Table S4: Comparison of in multiple logistic regression models including both chronological age and immune age metrics as predictors.

Odds ratios (95%-CI) from separate multiple logistic regression models fitted to the subsample of 547 complete observations with 199 events of low cardiorespiratory fitness (CRF) predicting the probability of low CRF by different immunosenescence biomarkers in addition to chronological age, with continuous predictors z-standardized to zero mean and unit variance, and adjusting the analyses for sex, obesity (BMI category) and physical activity level. For model comparison, Akaike's information criterion (AIC) is reported with lower values indicating improved fit.

Predictor	Immunosenescence biomarker included as predictor					
	**IMMAX**	**log CD8 mem/naive**	**logit CD8 CD28-**	**log NK/T**	**log CD4 mem/naive**	**log CD4/CD8**
**Immunosenescence** *(standardized)*	1.29 * (1.03 – 1.62)	1.32 * (1.06 – 1.66)	1.18 (0.97 – 1.44)	1.06 (0.87 – 1.29)	1.03 (0.85 – 1.26)	0.98 (0.80 – 1.20)
**Age** *(standardized)*	1.03 (0.83 – 1.29)	1.01 (0.81 – 1.27)	1.11 (0.91 – 1.36)	1.16 (0.96 – 1.41)	1.16 (0.95 – 1.41)	1.18 (0.96 – 1.45)
**Sex**						
males vs females	0.93 (0.62 – 1.40)	0.95 (0.63 – 1.42)	1.00 (0.67 – 1.49)	1.01 (0.67 – 1.51)	1.03 (0.68 – 1.53)	1.04 (0.69 – 1.54)
**BMI category**						
overweight vs normal	2.04 ** (1.33 – 3.14)	2.06 ** (1.34 – 3.18)	1.98 ** (1.29 – 3.04)	1.96 ** (1.28 – 3.01)	1.97 ** (1.28 – 3.02)	1.96 ** (1.28 – 3.00)
obese vs normal	6.01 *** (3.45 – 10.73)	6.12 *** (3.52 – 10.94)	6.09 *** (3.50 – 10.85)	6.13 *** (3.53 – 10.91)	6.16 *** (3.55 – 10.97)	6.19 *** (3.57 – 11.03)
**Physical activity**						
still acceptable vs low	0.64 * (0.41 – 0.99)	0.64 * (0.41 – 0.99)	0.65 (0.42 – 1.00)	0.66 (0.43 – 1.01)	0.67 (0.43 – 1.02)	0.67 (0.43 – 1.02)
satisfactory vs low	0.43 ** (0.22 – 0.77)	0.42 ** (0.22 – 0.76)	0.43 ** (0.23 – 0.78)	0.43 ** (0.23 – 0.77)	0.42 ** (0.22 – 0.77)	0.42 ** (0.22 – 0.77)
high vs low	0.43 * (0.20 – 0.88)	0.43 * (0.20 – 0.88)	0.43 * (0.20 – 0.88)	0.44 * (0.20 – 0.90)	0.44 * (0.20 – 0.90)	0.44 * (0.20 – 0.90)
AIC	656.814	655.602	658.768	661.212	661.474	661.546

*Odds ratios (95%-CI); * P<.05; ** P<.01; *** P<.001*

Table S5: Comparison of different age metrics (chronological age and immunosenescence biomarkers) predicting low CRF in separate models.

Odds ratios (95%-CI) from multiple logistic regression models fitted to the subsample of 547 complete observations with 199 events of low cardiorespiratory fitness (CRF) predicting the probability of low CRF by chronological age in comparison to different immunosenescence biomarkers, with continuous predictors z-standardized to zero mean and unit variance, and adjusting the analyses for obesity (BMI category) and physical activity level. For model comparison, Akaike's information criterion (AIC) is reported with lower values indicating improved fit.

		**Immunosenescence biomarkers**					
Predictors	**Chronological age**	**IMMAX**	**log CD8 mem/naive**	**logit CD8 CD28-**	**log NK/T**	**log CD4 mem/naive**	**log CD4/CD8**
**Age metric** (standardized)	1.17 (0.97 – 1.42)	1.30 ** (1.07 – 1.57)	1.32 ** (1.10 – 1.60)	1.22 * (1.01 – 1.48)	1.08 (0.90 – 1.31)	1.08 (0.89 – 1.30)	1.04 (0.86 – 1.26)
**BMI category**							
overweight vs normal	1.97 ** (1.30 – 3.00)	2.03 *** (1.34 – 3.07)	2.04 *** (1.35 – 3.10)	2.05 *** (1.36 – 3.11)	2.07 *** (1.37 – 3.13)	2.08 *** (1.38 – 3.15)	2.08 *** (1.38 – 3.15)
obese vs normal	6.23 *** (3.62 – 11.01)	5.96 *** (3.45 – 10.56)	6.07 *** (3.52 – 10.73)	6.28 *** (3.65 – 11.08)	6.43 *** (3.74 – 11.34)	6.46 *** (3.76 – 11.39)	6.53 *** (3.80 – 11.52)
**Physical activity**							
still acceptable vs low	0.67 (0.43 – 1.03)	0.64 * (0.41 – 0.99)	0.64 * (0.41 – 0.99)	0.66 (0.43 – 1.02)	0.68 (0.44 – 1.04)	0.69 (0.45 – 1.05)	0.69 (0.45 – 1.06)
satisfactory vs low	0.43 ** (0.23 – 0.77)	0.42 ** (0.22 – 0.76)	0.41 ** (0.22 – 0.75)	0.43 ** (0.23 – 0.78)	0.43 ** (0.23 – 0.78)	0.43 ** (0.23 – 0.77)	0.43 ** (0.23 – 0.78)
high vs low	0.44 * (0.20 – 0.90)	0.43 * (0.19 – 0.87)	0.43 * (0.19 – 0.87)	0.44 * (0.20 – 0.89)	0.45 * (0.21 – 0.92)	0.46 * (0.21 – 0.92)	0.46 * (0.21 – 0.93)
AIC	657.609	653.022	651.678	655.881	659.500	659.602	660.021

*Odds Ratios (95%-CI); * P<.05 ** P<.01 *** P<.001*

2 Supplemental Figures

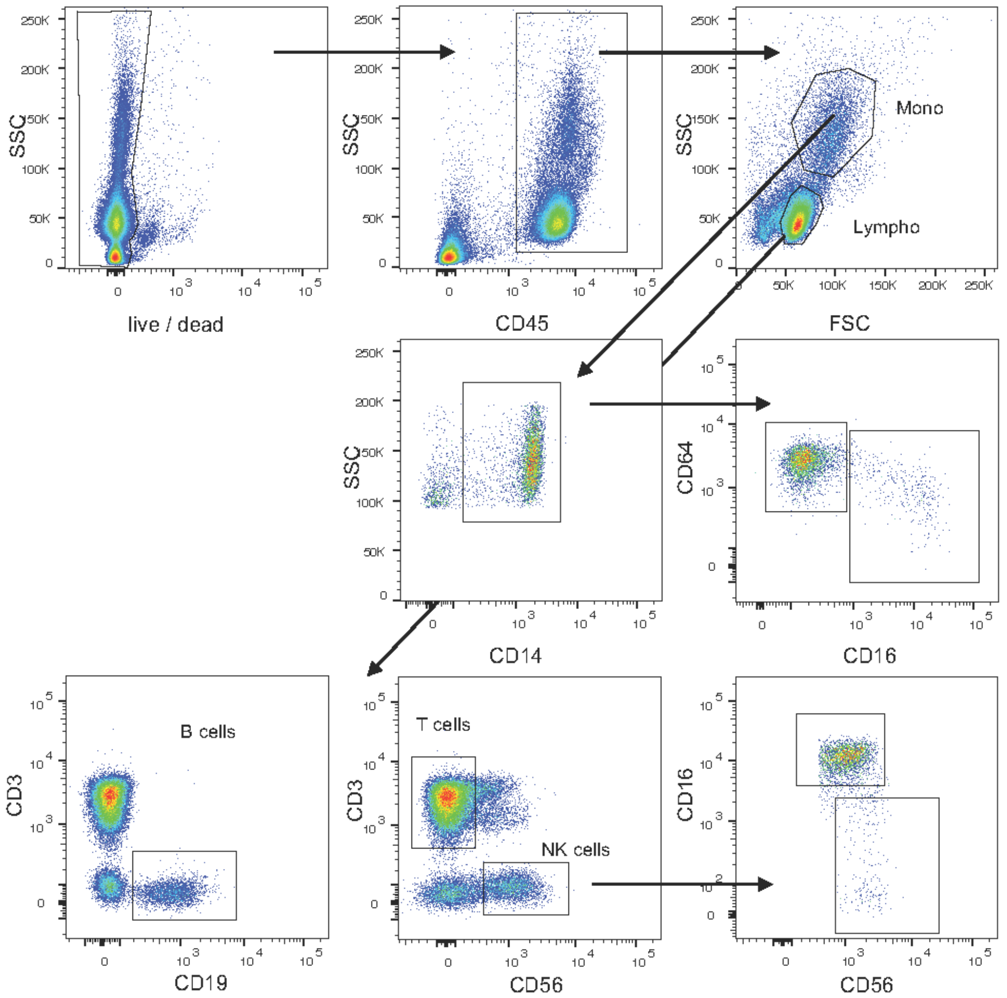

Figure S1: Gating strategy with general overview of lymphocyte and monocyte subpopulations

General overview of lymphocyte and monocyte subpopulations. Lymphocytes and monocytes are identified by CD45 and SSC characteristics. Based on CD14 gating, non-classical $CD16^+$ and classical $CD64^+$ monocytes can be identified. Lymphocytes were further sub-gated to identify B cells ($CD19^+$), T cells ($CD3^+CD56^–$), NK cells ($CD56^+CD3^–$) and $CD16^+$ $CD56^{dim}$ and $CD16^-$ $CD56^{bright}$ NK cells.

.

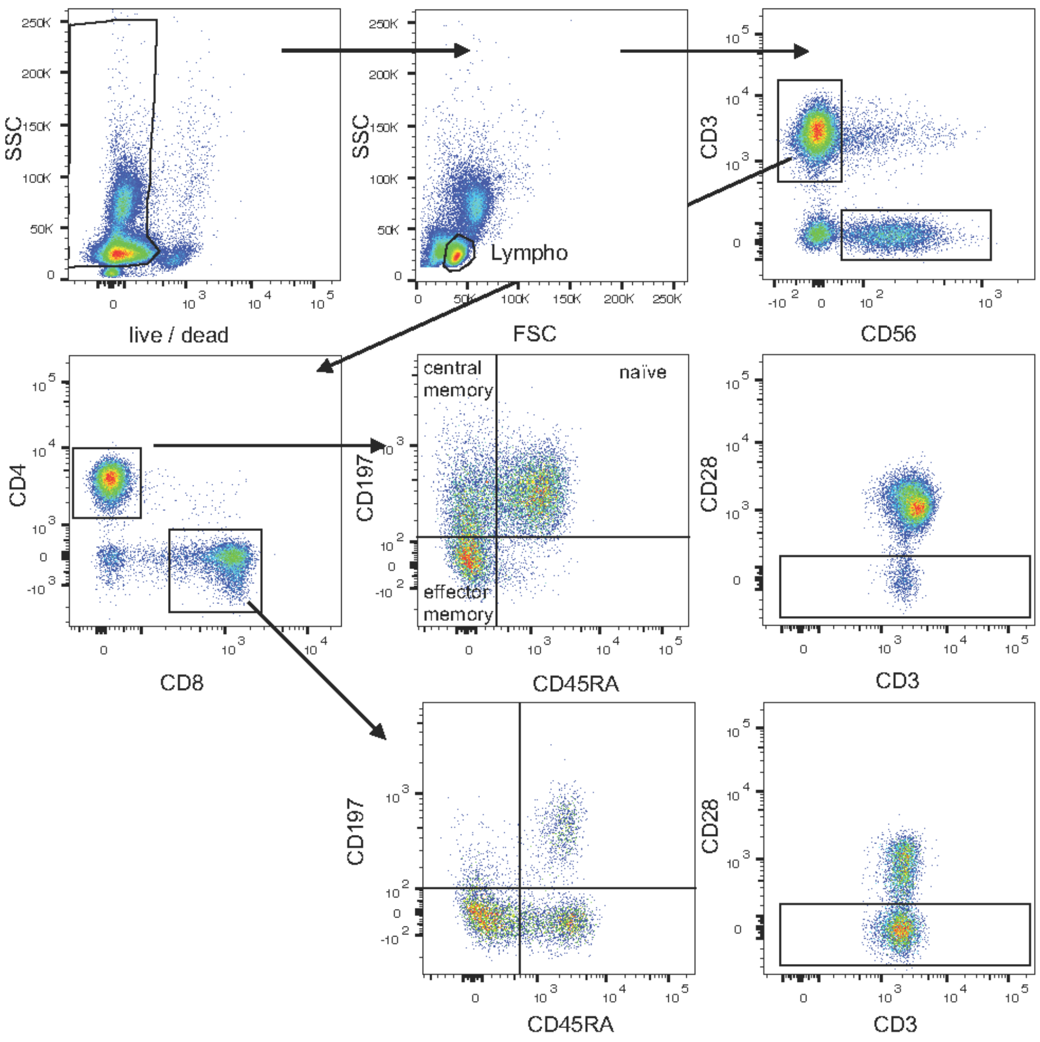

Figure S2: Gating strategy for T cell subpopulations.

NK and T cells were separated by CD3 and CD56. T helper cells and cytotoxic T cells were identified by CD4 and CD8 expression. Naïve, central and effector memory cells were separated within CD8 and CD4 T cells by CD45RA and CD197 staining. $CD28^{-}$ cells were sub-gated from CD8 and CD4 T cells.

.

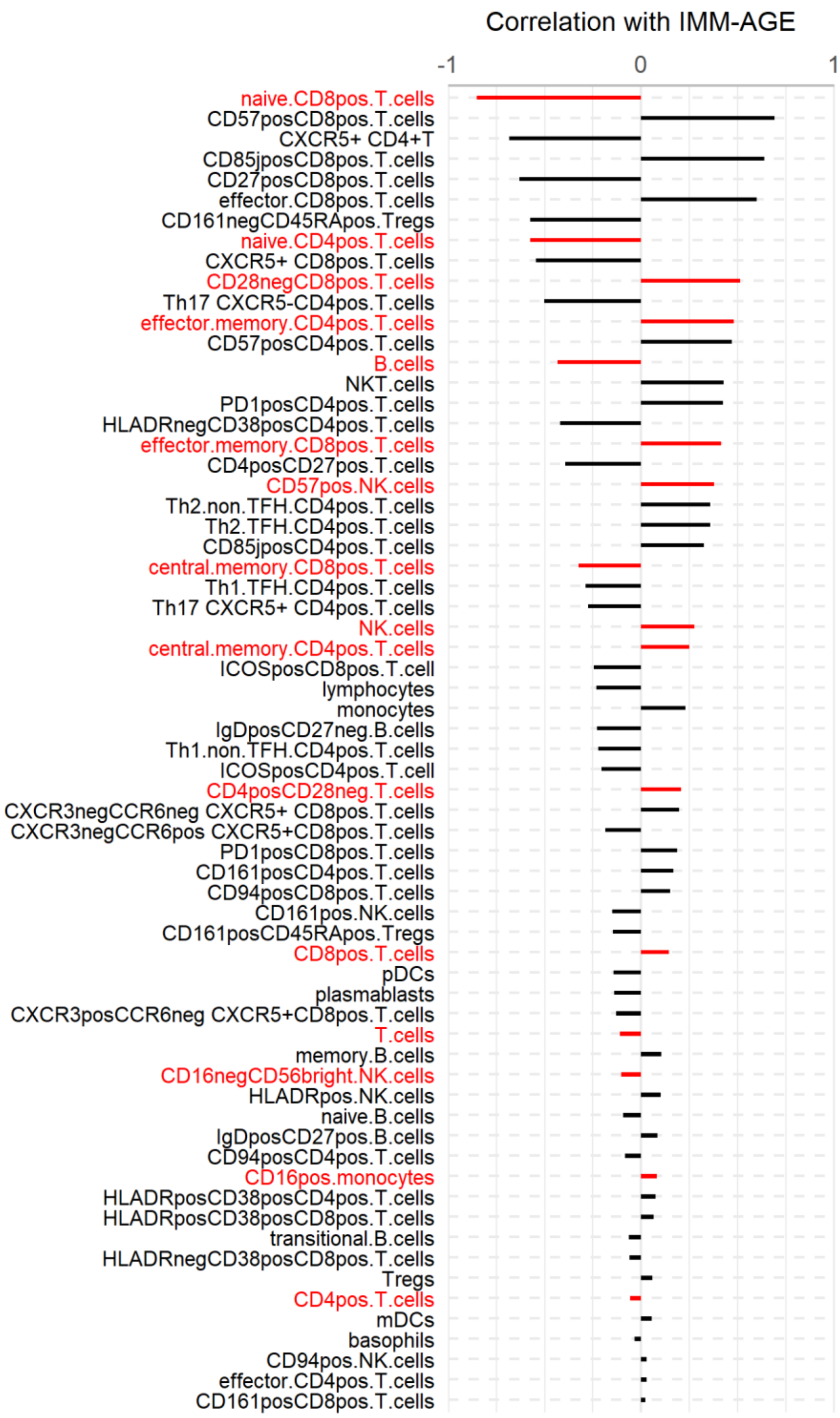

Figure S3: Correlation between IMM-AGE and 65 relative cellular frequencies

Bar chart of Pearson's correlation coefficients ordered by absolute values for the original 434 IMM-AGE observations (Alpert et al. 2019) related to cellular frequency data for 65 variables with bars and entries outlined in red indicating candidate variables potentially shared with the DVS.

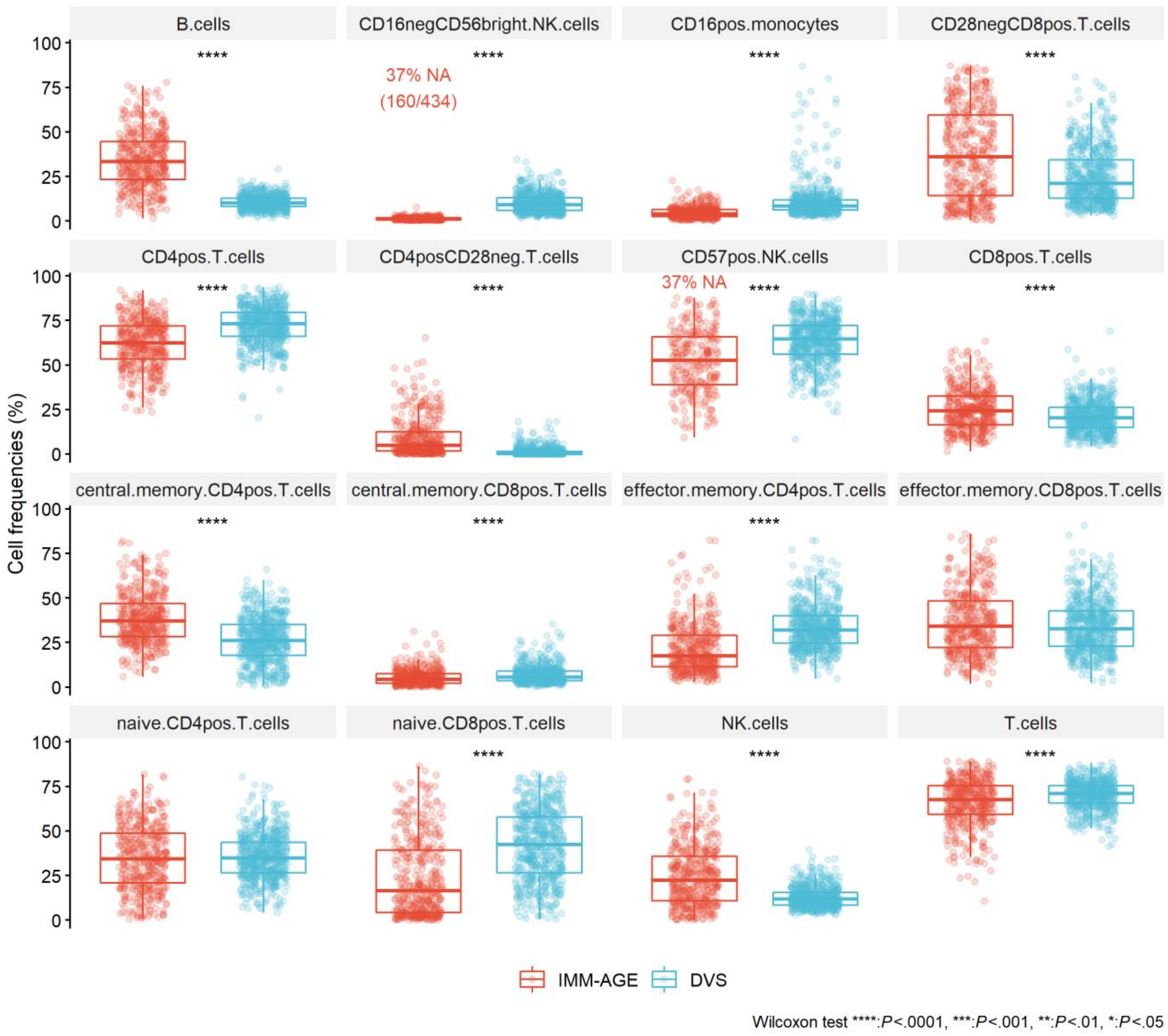

Figure S4: Comparison of 16 cellular frequencies between the DVS and IMM-AGE study

Box plots and Wilcoxon test results comparing the DVS data to the distributions of 16 shared (out of 65, cf. Table S2) raw cellular frequencies reported as percentages supplemental to the original IMM-AGE data (Alpert et al. 2019). Note the high number of missing values (NA) for the CD56-bright and CD57pos NK cells in the original study.

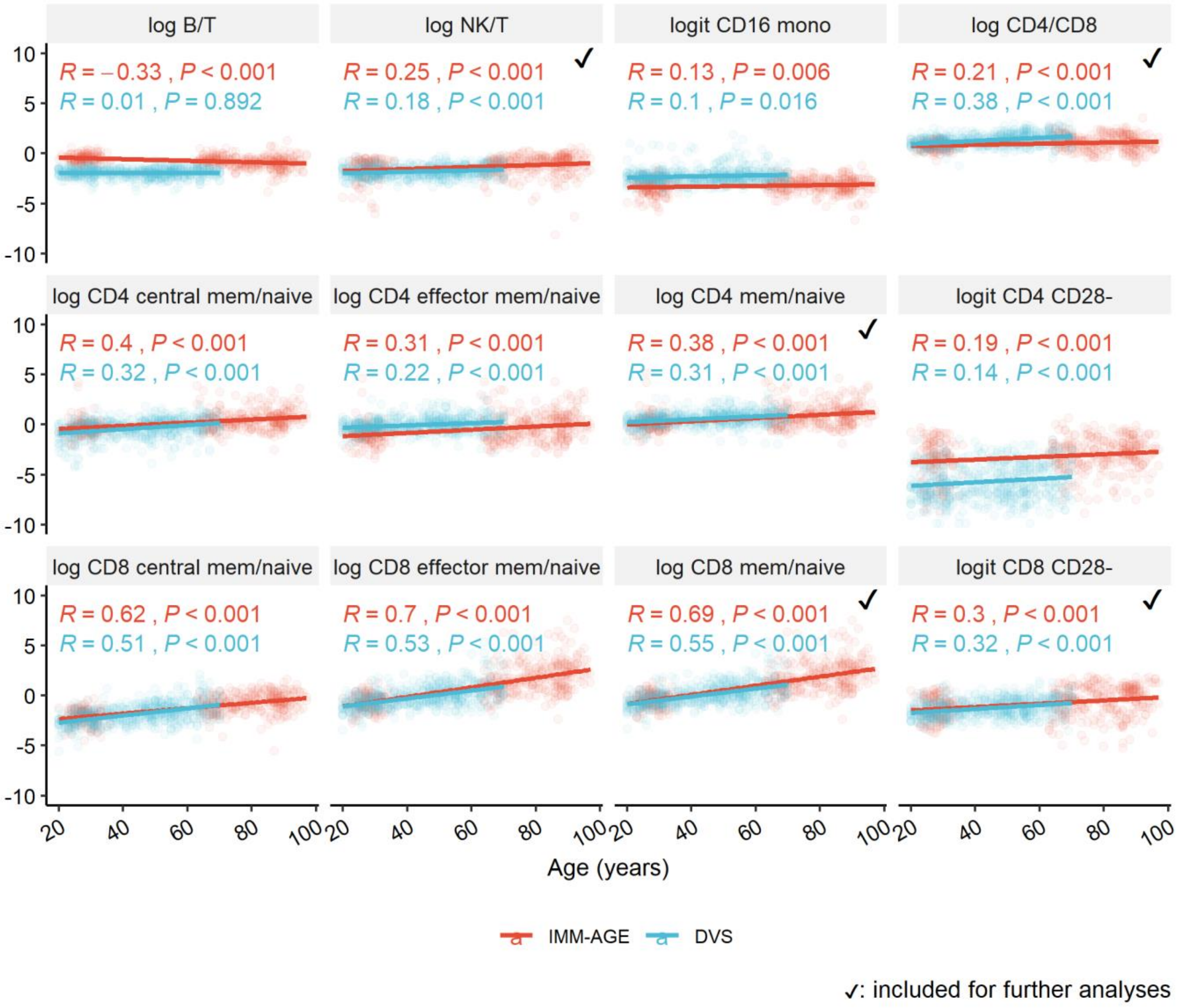

Figure S5: Comparison of linear regression lines for twelve candidate predictors related to age between the DVS and IMM-AGE study

Linear regression lines with Pearson correlation coefficients (R) for twelve candidate predictors related to age in the DVS compared to the original IMM-AGE study representing log-transformed ratios for compositional data and logit-transformed percentage cell frequencies. Check marks indicate predictors included in the final analysis shown in Figure 1.

3 Supplemental Code

Code S1: R script accessing IMM-AGE data and calculating IMMAX.

```
library(tidyverse)

## -- Attaching packages --------------------------------------- tidyverse 1.3.1 --

## v ggplot2 3.3.5     v purrr   0.3.4
## v tibble  3.1.6     v dplyr   1.0.8
## v tidyr   1.2.0     v stringr 1.4.0
## v readr   2.1.2     v forcats 0.5.1

## -- Conflicts ------------------------------------------ tidyverse_conflicts() --
## x dplyr::filter() masks stats::filter()
## x dplyr::lag()    masks stats::lag()

# read Supplemental Tables from Alpert et al. (2019)
library(readxl)
url1<-"https://static-content.springer.com/esm/art%3A10.1038%2Fs41591-019-0381-y/MediaOb
jects/41591_2019_381_MOESM3_ESM.xlsx"
tf <- tempfile(fileext = ".xlsx")
curl::curl_download(url1, tf)
# cell frequency data in sTable 5 of Alpert et al. (2019)
Alpert.cell.frequencies <- read_excel(tf, sheet=5L,skip=2,col_types = "numeric",na=c("",
"NA"))
# IMM-AGE values in sTable 11 of Alpert et al. (2019)
Alpert.index <- read_excel(tf, sheet=11L,skip=2,col_types = "numeric",na=c("","NA"))
unlink(tf)

Alpert.cell.frequencies$age<-NULL # do not overwrite age in Alpert.index
# list of raw cell frequency variables in Table S2
names(Alpert.cell.frequencies[-(1:3)])

##  [1] "basophils"
##  [2] "B.cells"
##  [3] "CD161negCD45RApos.Tregs"
##  [4] "CD161posCD45RApos.Tregs"
##  [5] "CD161posCD4pos.T.cells"
##  [6] "CD161posCD8pos.T.cells"
##  [7] "CD161pos.NK.cells"
##  [8] "CD16negCD56bright.NK.cells"
##  [9] "CD16pos.monocytes"
## [10] "CD27posCD8pos.T.cells"
## [11] "CD28negCD8pos.T.cells"
## [12] "CD4posCD27pos.T.cells"
## [13] "CD4posCD28neg.T.cells"
## [14] "CD4pos.T.cells"
## [15] "CD57posCD4pos.T.cells"
## [16] "CD57posCD8pos.T.cells"
## [17] "CD57pos.NK.cells"
## [18] "CD85jposCD4pos.T.cells"
## [19] "CD85jposCD8pos.T.cells"
## [20] "CD8pos.T.cells"
## [21] "CD94posCD4pos.T.cells"
## [22] "CD94posCD8pos.T.cells"
## [23] "CD94pos.NK.cells"
## [24] "central.memory.CD4pos.T.cells"
## [25] "central.memory.CD8pos.T.cells"
```

```
## [26] "effector.CD4pos.T.cells"
## [27] "effector.CD8pos.T.cells"
## [28] "effector.memory.CD4pos.T.cells"
## [29] "effector.memory.CD8pos.T.cells"
## [30] "HLADRnegCD38posCD4pos.T.cells"
## [31] "HLADRposCD38posCD4pos.T.cells"
## [32] "HLADRnegCD38posCD8pos.T.cells"
## [33] "HLADRposCD38posCD8pos.T.cells"
## [34] "HLADRpos.NK.cells"
## [35] "ICOSposCD4pos.T.cell"
## [36] "ICOSposCD8pos.T.cell"
## [37] "IgDposCD27neg.B.cells"
## [38] "IgDposCD27pos.B.cells"
## [39] "lymphocytes"
## [40] "mDCs"
## [41] "memory.B.cells"
## [42] "monocytes"
## [43] "naive.B.cells"
## [44] "naive.CD4pos.T.cells"
## [45] "naive.CD8pos.T.cells"
## [46] "NK.cells"
## [47] "NKT.cells"
## [48] "PD1posCD4pos.T.cells"
## [49] "PD1posCD8pos.T.cells"
## [50] "pDCs"
## [51] "plasmablasts"
## [52] "T.cells"
## [53] "CXCR5+ CD4+T"
## [54] "CXCR5+ CD8pos.T.cells"
## [55] "Th17 CXCR5-CD4pos.T.cells"
## [56] "Th17 CXCR5+ CD4pos.T.cells"
## [57] "CXCR3negCCR6pos CXCR5+CD8pos.T.cells"
## [58] "Th1.non.TFH.CD4pos.T.cells"
## [59] "Th1.TFH.CD4pos.T.cells"
## [60] "CXCR3posCCR6neg CXCR5+CD8pos.T.cells"
## [61] "Th2.non.TFH.CD4pos.T.cells"
## [62] "Th2.TFH.CD4pos.T.cells"
## [63] "CXCR3negCCR6neg CXCR5+ CD8pos.T.cells"
## [64] "transitional.B.cells"
## [65] "Tregs"

# merge cell frequencies with IMM-AGE metric
Alpert.data<-merge(Alpert.index,Alpert.cell.frequencies)

# list of variables potentially compatible to the DVS
compatible<-c(
  "B.cells",
  "NK.cells",
  "T.cells",
  "CD16negCD56bright.NK.cells",
  "CD16pos.monocytes",
  "CD28negCD8pos.T.cells",
  "CD4posCD28neg.T.cells",
  "CD4pos.T.cells",
  "CD57pos.NK.cells",
  "CD8pos.T.cells",
  "central.memory.CD4pos.T.cells",
  "central.memory.CD8pos.T.cells",
```

```
  "effector.memory.CD4pos.T.cells",
  "effector.memory.CD8pos.T.cells",
  "naive.CD4pos.T.cells",
  "naive.CD8pos.T.cells"
)


# Supplemental Figure Figure S3
# Pearson's correlations of 65 relative cell frequencies with IMM-AGE
Alpert.cor<-Alpert.data[names(Alpert.data)[c(4,6:70)]]
cors<-cor(Alpert.cor,method = 'pearson',use="pairwise.complete.obs")
rp<-cors[-1,1] # select correlations with IMM-AGE
# build data frame for plotting
rp.names<-rownames(cors)[-1]
cor.IMM.AGE<-cbind.data.frame(rp.names,rp)
cor.IMM.AGE$abs.rp<-abs(cor.IMM.AGE$rp)
cor.IMM.AGE$compatible<-ifelse(cor.IMM.AGE$rp.names %in% compatible,T,F)
# sort by descending absolute value of Person's correlation with IMM-AGE
cor.IMM.AGE<-cor.IMM.AGE %>%
  arrange(desc(abs.rp))
# outline candidate variables in red
a <- ifelse(cor.IMM.AGE$compatible, "red", "black")
color.vector=rev(a)

correlations=as.matrix(t(cor.IMM.AGE[65:1,2,drop=F]))
# plot data
df0<-arrange(data.frame(rp=t(correlations),labs=colnames(correlations)),abs(rp))
df0<-data.frame(rp=t(correlations),labs=colnames(correlations),color=color.vector)
df0$labs <- factor(df0$labs, levels = df0$labs)
# plot Figure S3
SUP.Figure.S3<-ggplot(data=df0,aes(x=labs,y=rp,fill=color))+
  geom_col(width=0.2)+ coord_flip() +
  scale_fill_manual(values = c('black','red')) +
  scale_y_continuous(position = 'right',
                     #   name=expression(rho),
                     limits = c(-1,1),
                     breaks = seq(-1,1,1),
                     minor_breaks = seq(-1,1,.25),
                     expand = expansion()
  ) +
  labs(title="Correlation with IMM-AGE")+
  theme_minimal() +
  theme(
    plot.title = element_text(hjust=0.5,size=8),
    axis.text.y = element_text(colour = color.vector,size=5),
    axis.text.x = element_text(size=6),
    axis.title.y = element_blank(),
    axis.title.x = element_blank(),#element_text(face = "italic"),
    axis.ticks.y = element_blank(),
    axis.line.x.top = element_line(colour = 'grey'),
    panel.grid.major.y = element_line(linetype=2),
    legend.position = "none"
  )

## Warning: Vectorized input to `element_text()` is not officially supported.
## Results may be unexpected or may change in future versions of ggplot2.
```

```
SUP.Figure.S3
```

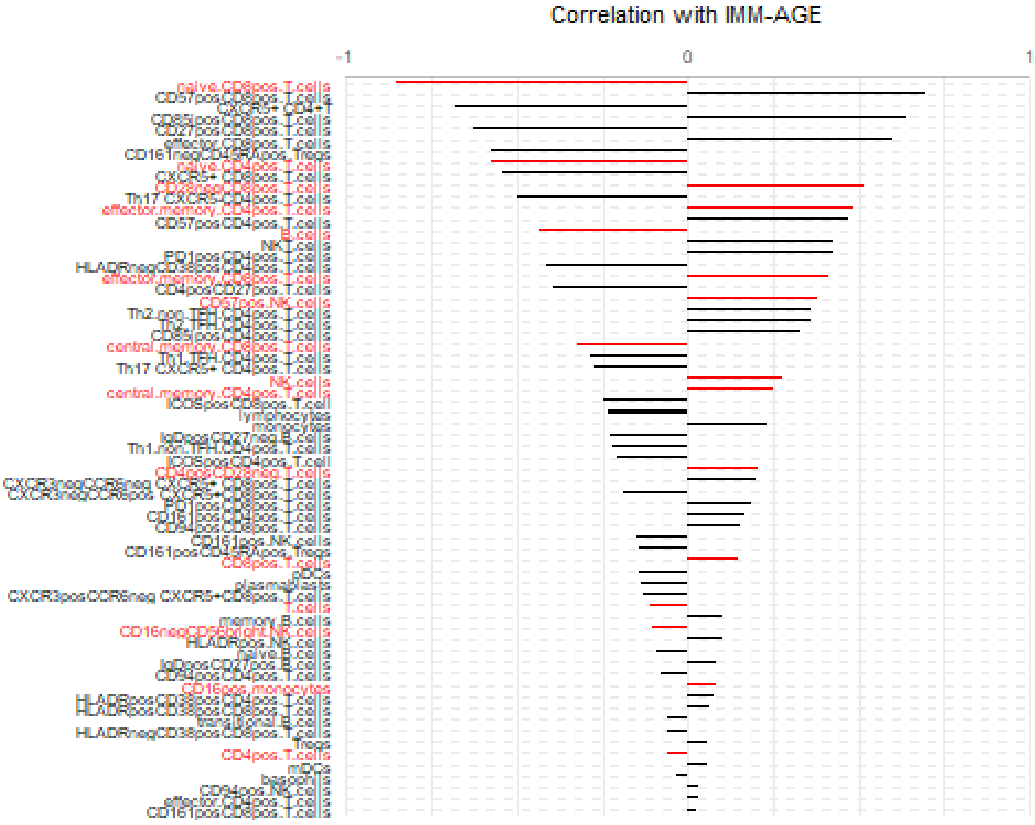


```
# Approximation of IMM-AGE by principal component analysis using package pls
library(pls)

##
## Attache Paket: 'pls'

## Das folgende Objekt ist maskiert 'package:stats':
##
##     loadings

# data frame with selected predictors
nam.pcr<-c("log.NK.T","log.T.CD4pos.CD8pos",
           "log.T.CD8.mem.naive","log.T.CD4.mem.naive",
           "logit.CD8posCD28neg.T.cells")
# calculate predictors
Alpert.data<-within(Alpert.data,
    {
     log.NK.T <- log(NK.cells/T.cells)
     logit.CD8posCD28neg.T.cells<-log(CD28negCD8pos.T.cells/(100-CD28negCD8pos.T.cells))
     log.T.CD4pos.CD8pos=log(CD4pos.T.cells/CD8pos.T.cells)
     log.T.CD4.mem.naive=log((central.memory.CD4pos.T.cells+effector.memory.CD4pos.T.cel
ls)/naive.CD4pos.T.cells)
     log.T.CD8.mem.naive=log((central.memory.CD8pos.T.cells+effector.memory.CD8pos.T.cel
ls)/naive.CD8pos.T.cells)
     }
)
# reduced data for fitting
Alpert.index.pcr<-Alpert.data[,which(names(Alpert.data) %in% nam.pcr)]
# fit to logit-transformed IMM-AGE ranging between 0 and 1 (including the limits)
```

```
# Function to transform rates for beta regression
# because values of 0 and/or 1 may occur, cf.
# Smithson, M., Verkuilen, J., 2006.
# A better lemon squeezer? Maximum-likelihood regression with beta-distributed dependent
variables.
# Psychological Methods 11, 54-71, https://doi.org/10.1037/1082-989X.11.1.54
transform_rate <- function(rate) {
  (rate * (length(rate) - 1) + 0.5)/length(rate)
}
Alpert.index.pcr$y = transform_rate(Alpert.data$`IMM-AGE`)
Alpert.index.pcr$logit.IMM.AGE<-log(Alpert.index.pcr$y/(1-Alpert.index.pcr$y))
Alpert.index.pcr$y<-NULL
# for reproducibility initialize random number generator
set.seed (1000)
pcr.model <- pcr(logit.IMM.AGE~., data = Alpert.index.pcr, scale = TRUE, validation = "C
V")
# Model summary
summary(pcr.model)

## Data:    X dimension: 434 5
##  Y dimension: 434 1
## Fit method: svdpc
## Number of components considered: 5
##
## VALIDATION: RMSEP
## Cross-validated using 10 random segments.
##        (Intercept)  1 comps  2 comps  3 comps  4 comps  5 comps
## CV          0.9516   0.6923   0.6742   0.6468   0.6247   0.6254
## adjCV       0.9516   0.6906   0.6738   0.6463   0.6241   0.6249
##
## TRAINING: % variance explained
##                 1 comps  2 comps  3 comps  4 comps  5 comps
## X                 33.66    59.72    79.04    93.80   100.00
## logit.IMM.AGE     47.51    50.45    54.41    58.05    58.07

# note that 4-component model yields lowest prediction error
# therefore, use this model for prediction and calculation of IMMAX
Alpert.data$IMMAX<-plogis(predict(pcr.model,ncomp=4,newdata = Alpert.data))
# Table S3: coefficients from principal component regression
betas<-coef(pcr.model,ncomp=4,intercept = T)
beta<-as.vector(betas)
predictors<-rownames(betas)
beta.df<-cbind.data.frame(predictors,beta)
# Alpert sample SD for scaled predictors
sds<-sapply(Alpert.index.pcr[nam.pcr],sd)
SD<-as.vector(sds)
predictors<-names(sds)
sd.df<-cbind.data.frame(predictors,SD)

# print Table S3
SUP.Table.S1<-merge(beta.df,sd.df,all.x = T)
SUP.Table.S1$i<-seq(0,5,1)
SUP.Table.S1<-SUP.Table.S1 %>%
  mutate(beta.SD=beta/SD)  %>%
  relocate(predictors,i,beta,SD,beta.SD)
colnam<-c("Predictor x[i]","i","coefficients beta[i]","SD[i]","beta[i] / SD[i]")
SUP.Table.S1
```

```
##                      predictors i       beta        SD     beta.SD
## 1                   (Intercept) 0 0.02479763        NA          NA
## 2                      log.NK.T 1 0.16234461 1.0539578 0.15403330
## 3           log.T.CD4.mem.naive 2 0.25494849 1.0774117 0.23663052
## 4           log.T.CD4pos.CD8pos 3 0.04371916 0.6835501 0.06395897
## 5           log.T.CD8.mem.naive 4 0.39622466 1.6799502 0.23585500
## 6 logit.CD8posCD28neg.T.cells 5 0.32666790 1.4338448 0.22782655
```

```
# Figure 1c: Fit of IMMAX vs IMM-AGE for Alpert
Alpert.data$IMM.AGE<-Alpert.data$`IMM-AGE`
ggplotRegression <- function(fit){

  require(ggplot2)

  ggplot(fit$model, aes_string(x = names(fit$model)[2], y = names(fit$model)[1])) +
    geom_point(col = "#E64B35FF",alpha=0.2) + #"#E64B35FF"
    annotate("text", x=0.0,y=0.98,size=3.5,hjust=0,vjust=0.5,
             label= paste("R = ",signif(sqrt(summary(fit)$adj.r.squared), 2),
                          "\nRMSE =",signif(summary(fit)$sigma, 5))) +
    labs(x="IMMAX",y="IMM-AGE",
         title = "IMM-AGE approximation by\nprincipal component regression")
}
Fig1.c<-ggplotRegression(lm(IMM.AGE ~ IMMAX,
                            data = Alpert.data)) +
  #  xlim(0,1)+ylim(0,1)+
  geom_abline(intercept = 0, slope = 1,lty=2) +
  scale_x_continuous(limits = c(0,1),breaks = seq(0,1,.2)) +
  scale_y_continuous(limits = c(0,1),breaks = seq(0,1,.2)) +
  theme_classic(11) +
  theme(
    plot.title = element_text(size=10)
  )
Fig1.c
```

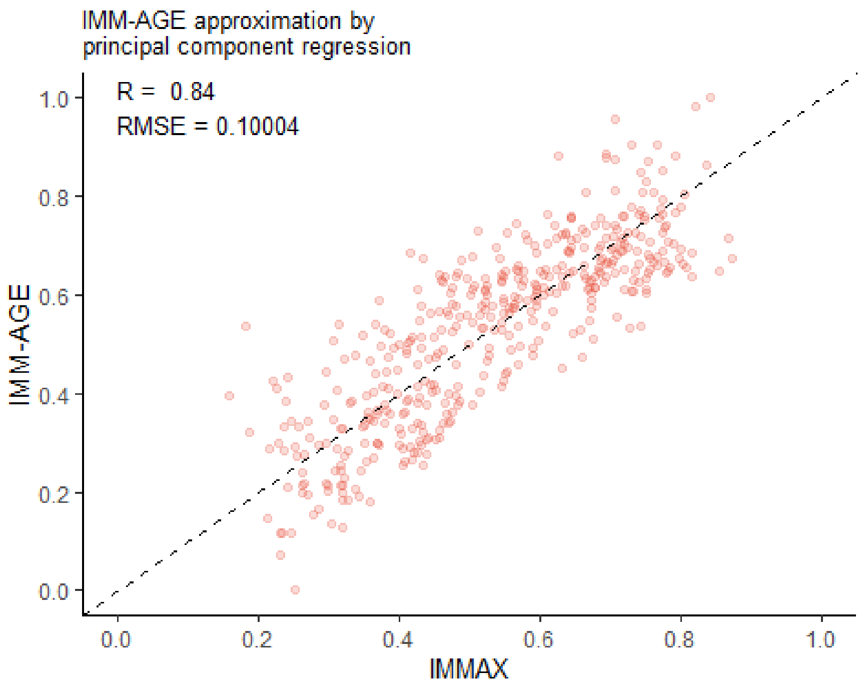

4 Abbreviations

#missing	number of missing observations
AIC	Akaike's information criterion
BMI	body-mass index
CD4	%CD4-positive T cells
CD8	%CD8-positive T cells
CD8 CD28-	%CD28-negative CD8-positive T cells
CI	Confidence interval
CRF	cardiorespiratory fitness
DVS	Dortmund Vital Study (ClinicalTrials.gov Identifier: NCT05155397)
ESM	Electronic supplemental material
FACS	Fluorescence-activated cell sorting
IMMAX	approximation to IMM-AGE metric (Alpert et al. 2019) by principal component regression, termed *Immune Age indeX*
log	natural logarithm
logit	transformation of a percentage (*%p*) by *logit(%p) = log(%p/(100%-%p))*
mem	%memory T cells
naïve	%naïve T cells
NK	%natural killer cells
OR	Odds Ratio
PBMC	Peripheral blood mononuclear cells
PWC130	power output from the physical working capacity test on the bicycle ergometer at 130 bpm standardized for body mass
R	Pearson's correlation coefficient
RMSE	root mean squared error
T	%T cells

5 References

Alpert A, Pickman Y, Leipold M, Rosenberg-Hasson Y, Ji X, Gaujoux R, Rabani H, Starosvetsky E, Kveler K, Schaffert S, Furman D, Caspi O, Rosenschein U, Khatri P, Dekker CL, Maecker HT, Davis MM, Shen-Orr SS (2019) A clinically meaningful metric of immune age derived from high-dimensional longitudinal monitoring. Nature Medicine 25 (3):487-495. https://doi.org/10.1038/s41591-019-0381-y
